# Kirsch’s, and everyone’s, bind: How to build models for the wild?

**DOI:** 10.1007/s10339-019-00914-1

**Published:** 2019-03-23

**Authors:** Konstantinos Katsikopoulos

**Affiliations:** Department of Decision Analytics and Risk, University of Southampton, Southampton, SO17 1BJ UK

**Keywords:** Cognitive modeling, Decision making, Heuristics

## Abstract

Alexandra Kirsch proposed a general formal model of decision making. She proposed it as a model both of human psychology and of artificial intelligence. As one might expect, and as Don Ross explicated, this is a challenging, albeit fascinating, position to occupy. In this comment, I sketch my own view of the bind and speculate on how to get out of it. In one sentence, my description of the bind is: How to build models for the wild? By models, I mean formal (mathematical, computer-based, precise conceptual) models, and by the wild, I mean “large worlds,” which are situations where uncertainty cannot be meaningfully reduced to well-developed devices such as probability. I discuss solutions for getting out of the bind proposed in the cognitive science of decision making. And I discuss how another discipline, operations research, has attempted to get out of the bind.

## Motivation and scope

Alexandra Kirsch (2019) proposed a general formal model of decision making. She proposed it as a model both of human psychology and of artificial intelligence (AI). As one might expect, and as Don Ross explicated (2019), this is a challenging, albeit fascinating, position to occupy. Notwithstanding all the lip service to interdisciplinary work, such endeavors still confuse people: Is Kirsch’s model descriptive (science) or prescriptive (engineering)? It seems that she says that it can be both, but isn’t it empirically unfalsifiable a la Popper for psychology purposes and too demanding to calibrate for AI? And, isn’t saying that the model is both descriptive and prescriptive tantamount to committing the naturalistic fallacy and identifying the *is* with the *ought*?

I believe that Kirsch’s approach is invaluable to the study of decision making. Kirsch invokes Herbert Simon’s (1969) *The Sciences of the Artificial*, and Ross argues that her work follows a similar approach. Indeed, Simon saw heuristics/computational shortcuts/simple rules of thumb as the stuff from which effective information processing—human or not—is made (Katsikopoulos and Lan 2011). Simon did not worry much about whether he did psychology, computer science, economics, engineering design or something else. He did all of the above because he saw that this was necessary in order to study decision making. He built theories of how people actually make decisions (psychology and economics) and turned them into algorithms for how an agent should make decisions (computer science and engineering design). It worked well. As we know, there is nothing as practical as a good theory; and while in theory there is no difference between theory and practice, in practice there is. Both theory and practice are indispensable for getting a handle on a difficult issue.

Did Simon do everything right and address all issues in decision research? Well, of course he couldn’t and of course he didn’t. (This is the man said to say that his papers—including the very many insightful and impactful ones he produced—were just work in progress.) If all research problems in decision making were solved, we reviewers of Kirsch’s article would not have been confused, and she would not have been in the bind that Ross identified and aptly noticed that all of us cognitive modelers are in.

In this comment, I sketch my own view of the bind and speculate on how to get out of it. In one sentence, my description of the bind is: *How to build models for the wild?* By *models*, I mean formal (mathematical, computer-based, precise conceptual) models, and by the *wild*, I mean “large worlds” (Savage 1954; Binmore 2009; Ross 2019), which are situations where uncertainty cannot be meaningfully reduced to well-developed devices such as probability.

I discuss this view in "Models for the wild in cognitive science?" section, with an emphasis on solutions for getting out of the bind proposed in the cognitive science of decision making. In "The case of operations research" section, I discuss how another discipline, *operations research* (OR), has attempted to get out of the bind. I hope that this discussion is worthwhile because the overlap and influence of OR with and on the social sciences are not appreciated or leveraged as much as it could be (see also Mirowski 1999). A brief summary of the comment is provided in "Summary" section.

## Models for the wild in cognitive science?

In his comment on Kirsch’s article, Ross delineates the methodologies of psychology and economics in studying decision making: Psychology is meant to study the actual heuristics used by people (as produced by natural selection and social pressures), whereas economics (neoclassical, although also behavioral; see Katsikopoulos 2014) is meant to start from the idea that human behavior is optimal and then builds formal models of optimization problems and derive their solutions. The economics approach is also used in computer science and engineering design.

Now, cognitive science is an interdisciplinary endeavor, incorporating psychology, computer science and engineering (and others such as anthropology). In many ways, cognitive science is heavily influenced by computer science and engineering, and has an emphasis on building models. On the other hand, unlike computer science and engineering, cognitive science does *not* really engage with the wild. In this sense, cognitive science does not acknowledge Kirsch’s bind. There are two aspects to this lack of engagement with the wild: (1) the data used to test cognitive science models and (2) the approach used to build cognitive models. I discuss these two aspects below.

When it comes to gathering empirical evidence, cognitive science is a traditional science and typically relies in the laboratory, which can provide experimental control. There have been attempts to understand “cognition in the wild” (Hutchins 1995) as well, with Gary Klein and colleagues’ research program on *naturalistic decision making* (Klein 2008) being a notable example, but they are the exception. The study of people’s classification processes is a case in point. Whereas in the early 1970s, psychologists travelled to Indonesian New Guinea to study how the indigenous Dani people classified colors and forms occurring in nature (Rosch 1973), subsequent classification research has by and large focused on how people learned to assign artificial stimuli into arbitrary-related classes in the controlled context of the laboratory (Katsikopoulos et al. 2019).

The disconnect with the wild is even more pronounced when it comes to building models. The models developed in cognitive science operate with way more information than what is typically available or desirable in the wild. Take, for example, the increasingly popular approach of Bayesian modeling (Chater and Oaksford 2008; Lee and Wagenmakers 2014). This approach requires prior probabilities as input, which by definition are hard to estimate reliably and validly in the wild. Furthermore, the approach outputs posterior probabilities,^1^ which are typically not what is needed in order to be effective in the wild: A medical doctor needs to *decide* whether a patient with intense chest pain is about to have a heart attack, and then needs to *act* and have a treatment implemented.

One might counter here that knowing the probability of the patient having a heart attack is useful, or even necessary, for deciding and acting. But this might be more of an academic mindset. Practicing emergency care doctors (Green and Mehr 1997), and all sorts of practitioners that work under demanding conditions, such as firefighters (Klein and Calderwood 1991) and checkpoint guards (Keller and Katsikopoulos 2016), do not seem to make decisions or take actions based on probabilities. A hard-nosed Bayesian might still insist that the practitioners *should* use probabilities to make decisions and take actions, but the evidence on the comparative accuracy and effectiveness of probability-based models and models that use simple, actionable rules does not support this conviction (for a review, see Katsikopoulos et al. 2019).

Notice that the above criticism of Bayesian cognitive science is distinct from other common criticisms, which are referring to non-falsifiability (Jones and Love 2011) or a focus on explaining outcomes after the fact (Glymour 2011), while neglecting the precise specification of the underlying processes (Brighton and Gigerenzer 2008). My point here is that Bayesian cognitive science does not seem to be fit for the wild, descriptively or prescriptively. Having said that, it should be acknowledged that some Bayesian cognitive research has attempted to show that (groups of) people can optimally judge probabilities of phenomena that do exist outside the laboratory, such as human life spans and box-office revenues of films (Griffiths and Tenenbaum 2006; for a methodological critique, however, see Eberhardt and Danks 2011). But this kind of cognition seems to be very different from what emergency care doctors, firefighters or checkpoint guards have to exercise in crucial and dangerous tasks.

In sum, in this section I argued that cognitive science has not really tried to build models for the wild. But this is not the only social science found wanting in this respect. Lo and Mueller (2010) complain about how economics models cannot grapple with uncertainty which cannot be reduced to probability. The authors ‘blame’ an attitude of over-reliance on the modeling approach of physics and of the natural sciences more generally. Each discipline, while it should definitely be informed by ideas in other disciplines—remember Simon?—must ultimately develop its own models, catering to its own unique needs. Recent cognitive science modeling has been too much influenced by the ideal of optimization. But optimization might not be the only interdisciplinary route to study decision making, as the following example illustrates.

## The case of operations research

Operations research (OR; Hillier and Lieberman 2001) is the study *par excellence* of prescriptive decision making; that is, how decisions should be made.^2^ Developed during World War II to support the Allied Forces’ efforts, OR has always been tasked to be ready for use. In fact, the acronym stands for *operational* research in the UK. Tellingly, the presidential address of Geoff Royston (2013) to the Operational Research Society was titled *Operational Research for the Real* World, akin to Savage’s large worlds. This task specification of OR has had a profound effect on how it attempts to build models for the wild.

A great number of optimization problems, which subsequently were studied in the cognitive laboratory, were originally studied mathematically in OR: These include sequential decision-making problems such as Markov decision processes, optimal stopping problems and multi-armed bandit problems. These problems were later studied in the mathematical psychology of the 1960s and eventually resurfaced in the cognitive science of the 2000s. (Just in order to not over-emphasize the impact of OR modeling on cognitive science, however, it must be recognized that other significant external modeling influences on cognitive science also exist, as from *machine learning*—especially in recent years—and more specifically from reinforcement learning and statistical learning theory.)

Now, because OR was tasked to be ready for use in the wild, it did not accept unquestioningly the *promise* of optimization. An optimized decision is optimal only with respect to the model it was derived from; this decision’s optimal, or just adequate performance, in the wild is an empirical question. Additionally, in OR, robustness has been a virtue sometimes valued more than optimality (Rosenhead et al. 1972).

Of course, the limitations of optimization were routinely overlooked even within OR, with sometimes disastrous consequences (Ackoff 1979). But there has always been a healthy skepticism toward optimization. *The Science of Better* (not of *Best*!) was and still remains a popular explication of what OR is. This skepticism was more pronounced in the arguably more pragmatic side of the pond (the UK; Mingers 2011). Over the last 50 years, the movement of *Soft* OR has offered rigorous and structured, but not over-mathematized, models suggesting how practitioners should make decisions in the wild. For example, *problem*-*structured* models are particularly popular in multi-criteria decision making (Belton and Stewart 2002). *Hard* OR, which primarily develops optimizing models, has also embraced techniques of heuristic search, for example in computationally intractable problems such as those appearing in transportation and logistics.

As said, OR models are meant to be prescriptive in the wild. Interestingly, they can also be descriptive. This is by definition: Some practitioners *are* trained in OR models—hard and soft ones—and then might well choose to use them. The ‘new’ sub-discipline of *Behavioral* OR (Kunc et al. 2016) attempts to study such situations.

In sum, operations research has built models for the wild. This is because its task description is to be ready for use in the wild. In some ways, operations research models might be more human than those developed in the human sciences, because they need to be *actually used* by humans. While such models could lack the precise specification of underlying processes that cognitive science should strive for, they might constitute good starting points for building cognitive models for the wild.

## Summary

Kirsch (2019) proposed a general formal model of decision making and Ross (2019) pointed out that her endeavor, and its reception by us reviewers, showed that cognitive modeling is in a bind. In my own words, the bind is: *How to build models for the wild?* Models are formal (mathematical, computer-based, precise conceptual) constructions, and the wild is situations where uncertainty cannot be reduced to probability. In this comment, I complained that the prominent social sciences, psychology, economics and cognitive science do not seem to be engaged in building models for the wild. Operations research, which by some accounts is also a social science, has at least tried. I argued that this might be because its pragmatic orientation encouraged it to look beyond the promise of optimization and create models usable by people. If we cognitive scientists today take seriously Herbert Simon’s example of studying decision making in an interdisciplinary way, then we should also try to do the same and move outside of our comfort zone.

## Footnotes

1 It has been argued that “Bayesian brains” can work “without probabilities” (Sanborn and Chater 2016). This means that probabilities can be estimated by sampling. This might be true but presupposes that uncertainty in the world can be represented by probabilities.

2 Interestingly, Hebert Simon sometimes wore the hat of an operations researcher. He has blended cognitive science, artificial intelligence, heuristics and operations research in a provocative article (Simon and Newell 1958).
